# *cis*-Acting Elements and *trans*-Acting Factors in the Transcriptional Regulation of Raf Kinase Inhibitory Protein Expression

**DOI:** 10.1371/journal.pone.0083097

**Published:** 2013-12-26

**Authors:** Boyan Zhang, Ou Wang, Jingchao Qin, Shuaishuai Liu, Sheng Sun, Huitu Liu, Jian Kuang, Guohua Jiang, Wei Zhang

**Affiliations:** 1 Key Laboratory of Cell Proliferation and Regulation Biology of Ministry of Education, College of Life Sciences, Beijing Normal University, Beijing, China; 2 Department of Experimental Therapeutics, The University of Texas MD Anderson Cancer Center, Houston, Texas, United States of America; 3 Analysis and Testing Center, Beijing Normal University, Beijing, China; University of Hong Kong, Hong Kong

## Abstract

The Raf kinase inhibitory protein (RKIP) is down-regulated in multiple types of human cancers. Decreased RKIP transcription activity may be one of the major mechanisms responsible for the downregulation of RKIP expression in human diseases. To test this hypothesis, we need to gain basic knowledge of the transcriptional regulation of RKIP. To achieve this objective, we made a systematic effort to identify *cis*-acting elements and *trans*-acting factors that control RKIP promoter activity. We found that full RKIP promoter activity requires the region −56 to +261 relative to the transcription start site. Within the full promoter region, there are two motifs rich in G/C that responded to transcription factor Sp1, one cAMP-responsive element that responded to the transcription factor CREB, and one docking site for the histone acetylase p300. In human melanoma A375 cells and human cervical cancer HeLa cells, mutation or deletion of each of these *cis*-acting elements decreased promoter activity. In A375 cells, knockdown of the corresponding transcription factors Sp1, CREB, or p300 decreased RKIP promoter activity, whereas overexpression of CREB and p300 increased RKIP promoter activity. The results obtained with HeLa cells also supported the idea that Sp1 and CREB play positive roles in the regulation of RKIP transcription. These findings suggest that regulators of the expression or activity of Sp1, CREB, and p300 are involved in regulating RKIP transcription.

## Introduction

Activation of receptor tyrosine kinases by growth factors and cytokines promotes cell proliferation, survival, and migration through activation of the Ras-Raf-MEK-ERK cascade [1], [2]. This signaling cascade is hyperactivated in human pathological processes, including cancer and Alzheimer’s disease [3], [4]. The hyperactivation of the Ras-Raf-MEK-ERK signaling cascade in human diseases can be caused by overexpression or overactivation of the positive regulators or by downregulation or inactivation of the negative regulators in this cascade. Multiple mechanisms have been identified to be responsible for the hyperactivation of the positive regulators [5], [6]. However, the mechanisms that are responsible for the downregulation or inactivation of the negative regulators are much less understood.

Raf kinase inhibitory protein (RKIP, also known as PEBP1) is a well-characterized inhibitor of Raf kinase [7]. It is downregulated in multiple types of human cancers [8]–[10], which results in the overactivation of MEK and ERK. RKIP downregulation is a frequent event in epithelial-to-mesenchymal transition, and it is associated with cancer metastasis and poor prognosis [11]–[13]. However, the mechanisms responsible for the downregulation of RKIP in human cancers are not well understood. Okita et al found evidence that the level of RKIP transcript is decreased in the hippocampi of autopsied brains of patients with Alzheimer’s disease compared with those of non-demented control subjects [14]. Thus, decreased RKIP transcription activity may be one of the major mechanisms responsible for the downregulation of RKIP expression in human diseases. To test this hypothesis, we need to gain basic knowledge of the transcriptional regulation of RKIP. To achieve this objective, we designed the current study to identify the *cis*-acting elements and the *trans*-acting factors that regulate RKIP promoter activity.

Luciferase-based reporter activity assay and electrophoretic mobility shift assay (EMSA) are well-established approaches to identifying *cis*-acting elements and *trans*-acting factors that regulate gene transcription [15]. Using these approaches, we defined an RKIP promoter region and identified three kinds of *cis*-acting elements and corresponding transcription factors that regulate RKIP promoter activity. Our results demonstrated, for the first time, that Sp1, CREB, and p300 are among the critical transcription factors that positively regulate RKIP transcription.

## Results

### Identification of the Promoter Region that Drives RKIP Transcription

**Figure 1:**
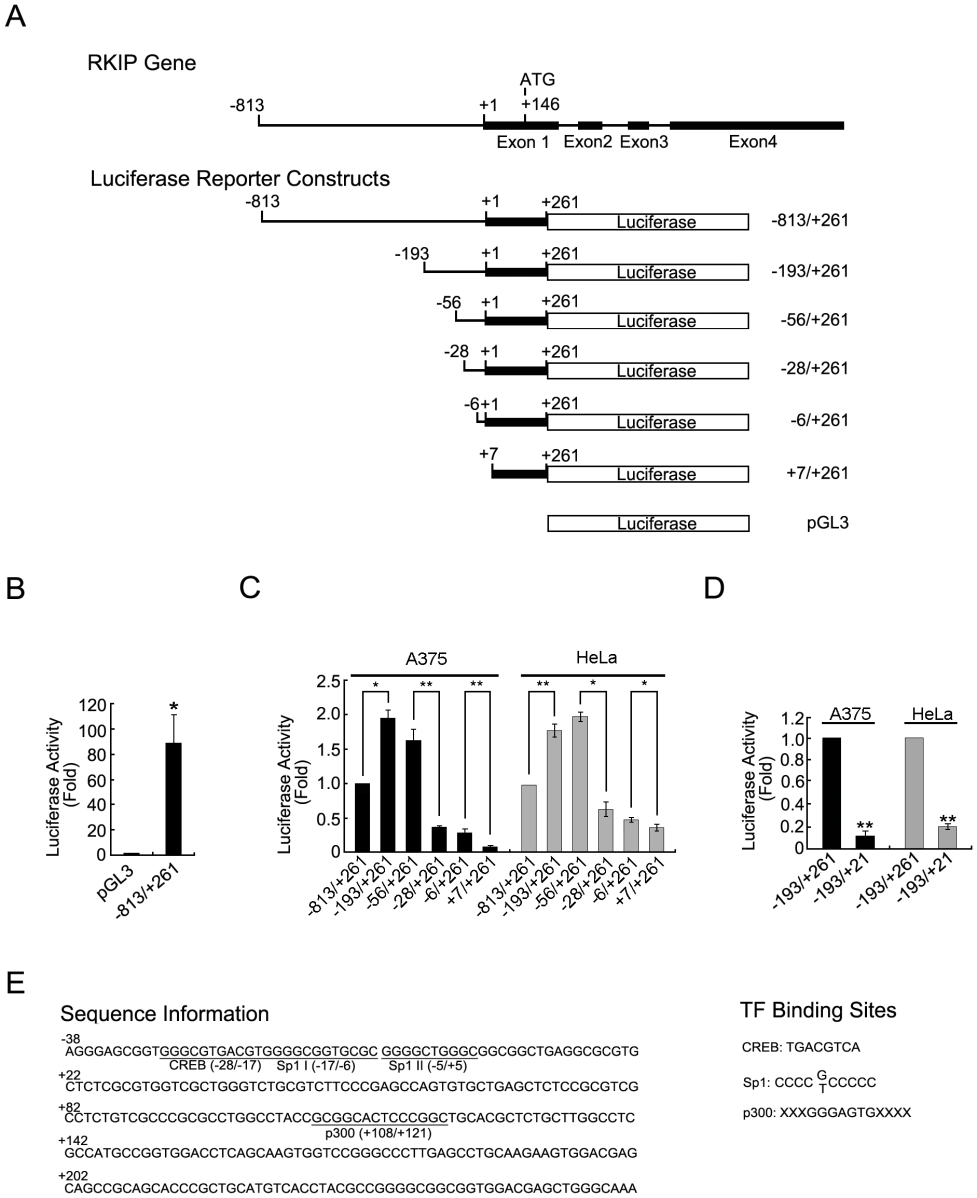
Identification of the promoter region of RKIP. (A) Schematic illustrations of the 5′ end portion of the RKIP gene structure and the starting reporter constructs used in this study. (B) Relative luciferase activity of A375 cells transfected with the pGL3-Basic RKIP (−813/+261) plasmid or the parental plasmid. Results of transient luciferase assays are shown in fold change relative to the pGL3-Basic vector. (C) Relative luciferase activity of A375 or HeLa cells transfected with different 5′ deletions of the pGL3-Basic RKIP (−813/+261) plasmid. Results of transient luciferase assays are shown in fold relative to pGL3-Basic RKIP (−813/+261). Significant differences between groups are as indicated. (D) Relative luciferase activity of A375 or HeLa cells transfected with the parental or a 3′ end-deleted pGL3-Basic RKIP (−813/+261) plasmid. (E) Four potential transcription factor binding sites present in region −56 to +261 region of the RKIP gene. *P<0.05, **P<0.01.

In a typical gene, the region −40 to +50 relative to a transcription start site constitutes the core promoter region in which the *Pol-*II-containing transcription machinery can be assembled [16]. Areas upstream and downstream of the core promoter region often contain sequences that regulate the rate of transcription that is driven by the assembled transcription machinery. Thus, to identify a full promoter region that supports RKIP transcription, we initially amplified the region −813 to +261 of the human RKIP gene and cloned it into a promoterless luciferase reporter plasmid (Fig. 1A). As determined by luciferase assays of human melanoma A375 cells transfected with this plasmid or the parental pGL3-Basic plasmid, only the plasmid with the inserted RKIP sequence produced significant luciferase activity (Fig. 1B), indicating that the inserted sequence contains potent promoter activity.

To further define the promoter region, we performed 5′- and 3′-deletions of the inserted RKIP sequence and determined the effect on its promoter activity by luciferase assay in A375 cells and in HeLa cells. From the 5′ end, deletion of the region −813 to −194 increased luciferase activity by ∼2-fold in both cell lines, whereas the next deletion (region −193 to −57) produced minor and inconsistent effects in these cell lines (Fig. 1C). However, an additional deletion of the region −56 to −7 caused a >3-fold reduction in the luciferase activity in both cell lines. Further deletions to the −6 or the +7 site caused further reductions in the luciferase activity in both cell lines (Fig. 1C). From the 3′ end, a single deletion of the region +22 to +261 caused a >3-fold reduction in luciferase activity in both cell lines (Fig. 1D). Collectively, these results indicate that the region of the inserted RKIP sequence from −56 to +261 is sufficient for full RKIP promoter activity and that the region −813 to −194 negatively regulates the promoter activity.

To identify putative *cis*-acting elements within region −56 to +261 of the inserted RKIP sequence, we searched the sequence for potential transcription factor binding sites. Although no TATA box sequence was identified, two putative Sp1 binding sites (−17 to −6 and −5 to +5), one putative CREB binding site (−28 to −17) and one p300 binding site (+108 to +121) were identified within this region (Fig. 1E). Thus, we speculated that these *cis*-acting elements and the corresponding transcription factors regulate RKIP transcription.

### Role of Sp1 Binding in Regulating RKIP Transcription

**Figure 2:**
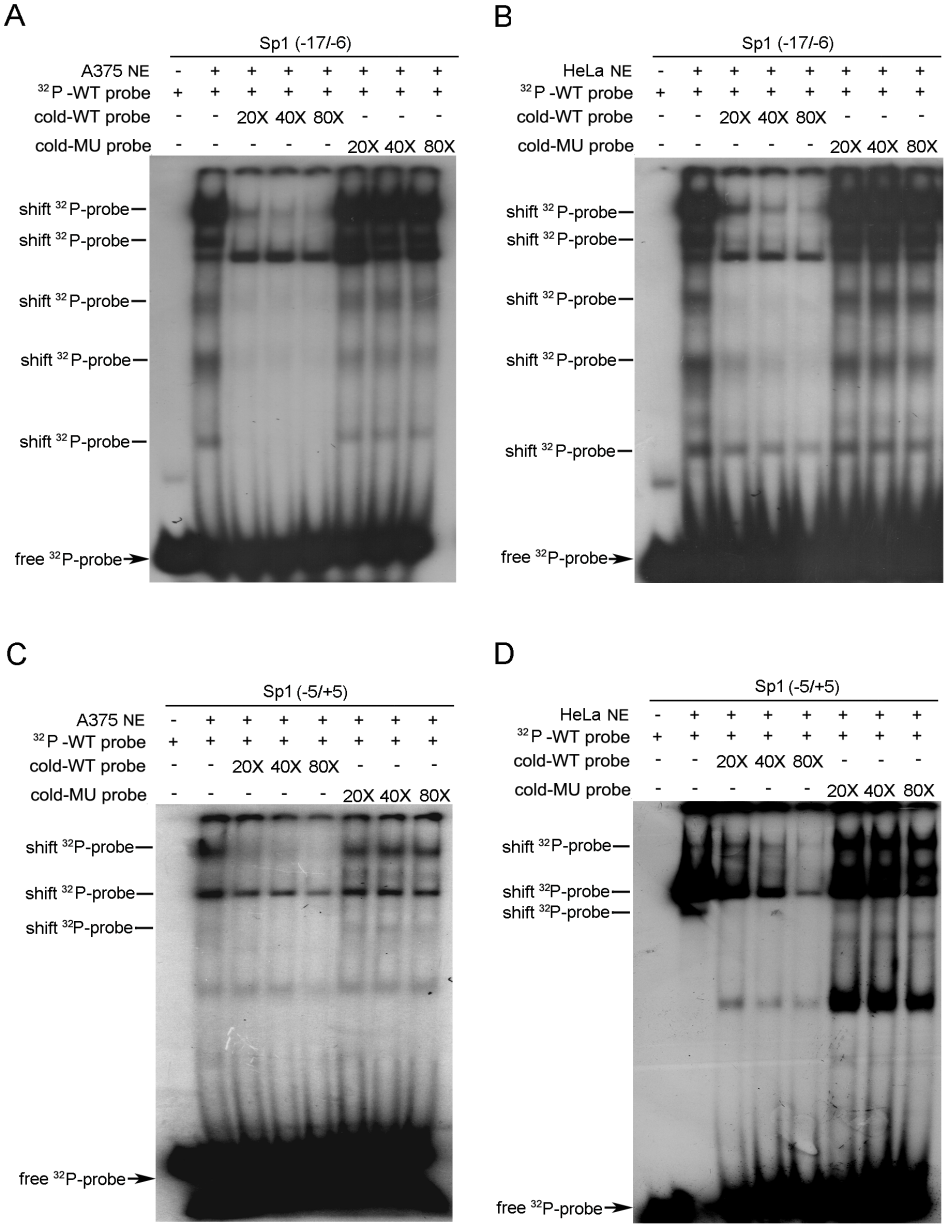
Oligonucleotides of Sp1 binding sites recognized by factors in A375 and HeLa cells. Shifted ^32^P-probe bands sensitive to competition by the WT cold probe are indicated. In A375 (A) and HeLa (B) nuclear extracts, EMSA of the ^32^P-labeled oligonucleotide derived from the −17/−6 putative Sp1 binding site in the presence or absence of cold WT or mutation oligonucleotide. In A375 (C) and HeLa (D) nuclear extracts, EMSA of the ^32^P-labeled oligonucleotide derived from the −5/+5 putative Sp1 binding site in the presence or absence of cold WT or mutation oligonucleotide. MU, mutated; NE, nuclear extract.

For a TATA-less promoter, binding of Sp1 to a site or sites close to the transcription start site often plays a key role in the assembly of a functional transcription machinery [17]–[19]. To define the role of the two putative Sp1 binding sites in RKIP promoter activity, we synthesized two pairs of oligonucleotides corresponding to the two Sp1 binding site sequences and determined their recognition by factors in nuclear extracts of A375 or HeLa cells by EMSA. Incubation of the ^32^P-labeled oligonucleotide derived from either the −17 to −6 site (Sp1 I) or the −5 to +5 site (Sp1 II) with nuclear extracts of A375 or HeLa cells resulted in multiple electrophoretic mobility shifts (Fig. 2). In each case, adding a great excess (20-, 40-, or 80-fold) of the cold wild-type (WT) oligonucleotide inhibited the shifts in a dose-dependent manner, whereas adding the same concentrations of the mutant oligonucleotide did not have notable inhibitory effects. These results indicate that both of the putative Sp1 binding sites contribute to RKIP promoter activity.

**Figure 3:**
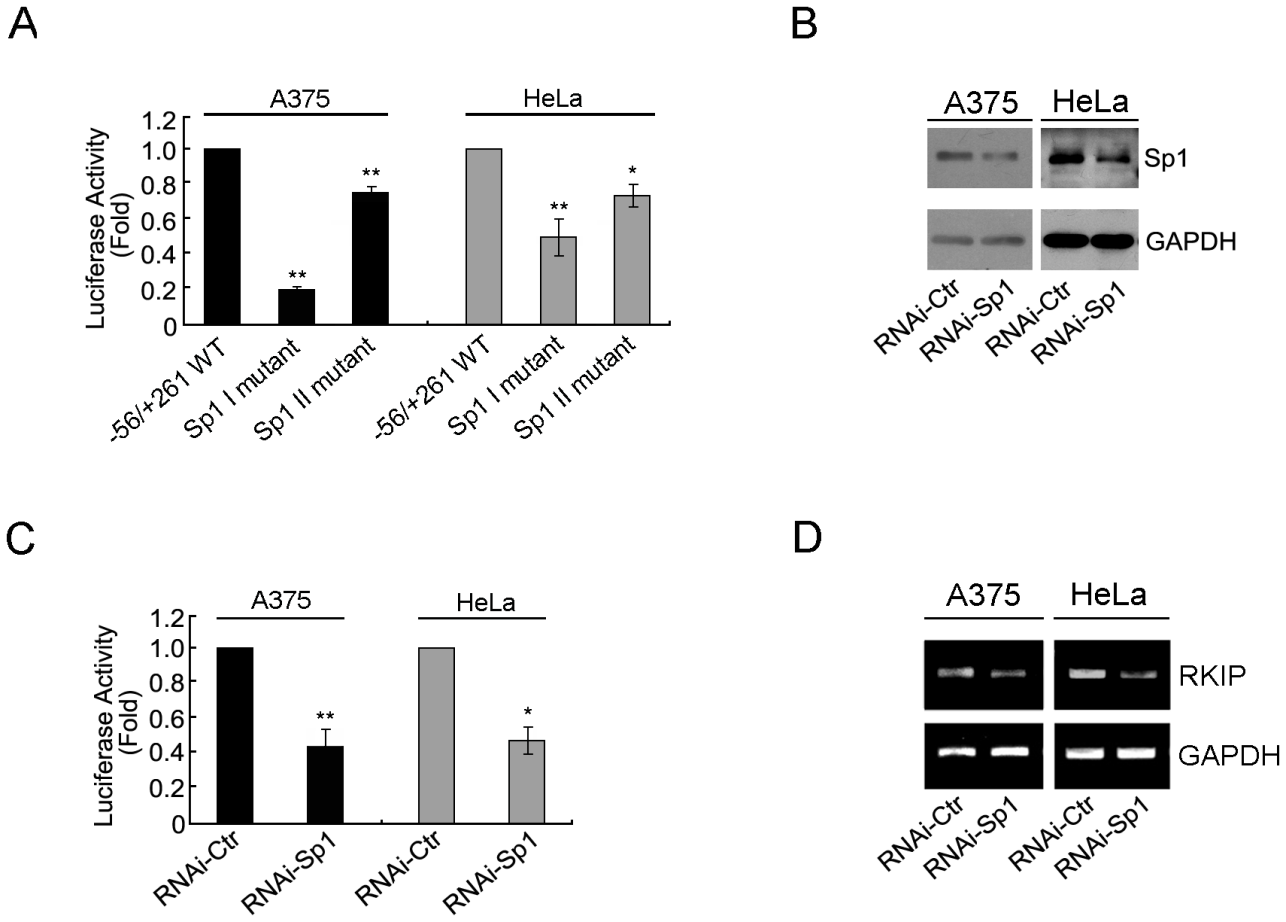
Role of Sp1 binding in regulating RKIP promoter activity in A375 and HeLa cells. (A) Relative luciferase activity of cells transfected with each of the indicated reporter constructs. (B) Cells were transfected with the indicated siRNAs and cultured for an additional 48 hr, then the expression levels of Sp1 and GAPDH were determined by immunoblotting. (C) Cells were co-transfected with the indicated siRNAs and the reporter construct pGL3-Basic RKIP (−56/+261) plasmid and then cultured for additional 48 hr. Relative luciferase activity was assessed by luciferase assay. (D) Cells were co-transfected with the indicated siRNAs and then the mRNA level of RKIP was examined by RT-PCR. *P<0.05, **P<0.01.

To test this hypothesis, we mutated these two Sp1 binding sites in the reporter construct containing the region −56 to +261 and determined the effect on promoter activity in transfected A375 or HeLa cells. Mutation of the Sp1 I or Sp1 II region reduced RKIP promoter activity in A375 cells (by ∼80% and ∼25%, respectively) and in HeLa cells (by ∼55% and ∼25%, respectively) (Fig. 3A). These results demonstrate that both Sp1 binding sites are important for RKIP promoter activity.

The critical roles of these two Sp1 binding sites in RKIP promoter activity indicate that Sp1 plays a positive role in RKIP transcription. To test this prediction, we transfected A375 or HeLa cells with Sp1-specific siRNA or negative control siRNA and determined the effect of Sp1 knockdown on both the promoter activity of the reporter construct and the level of endogenous RKIP transcript. In both cell lines, as determined by immunoblotting, transfection with Sp1-specific siRNA reduced the expression of Sp1 to ∼50% of its original level (Fig. 3B). This level of Sp1 knockdown resulted in an ∼50% reduction in the promoter activity (Fig. 3C) and a similar reduction in the RKIP transcript level (Fig. 3D). Taken together, these results support our hypothesis that Sp1 plays a positive role in regulating RKIP transcription.

### Role of CREB Binding in Regulating RKIP Transcription

**Figure 4:**
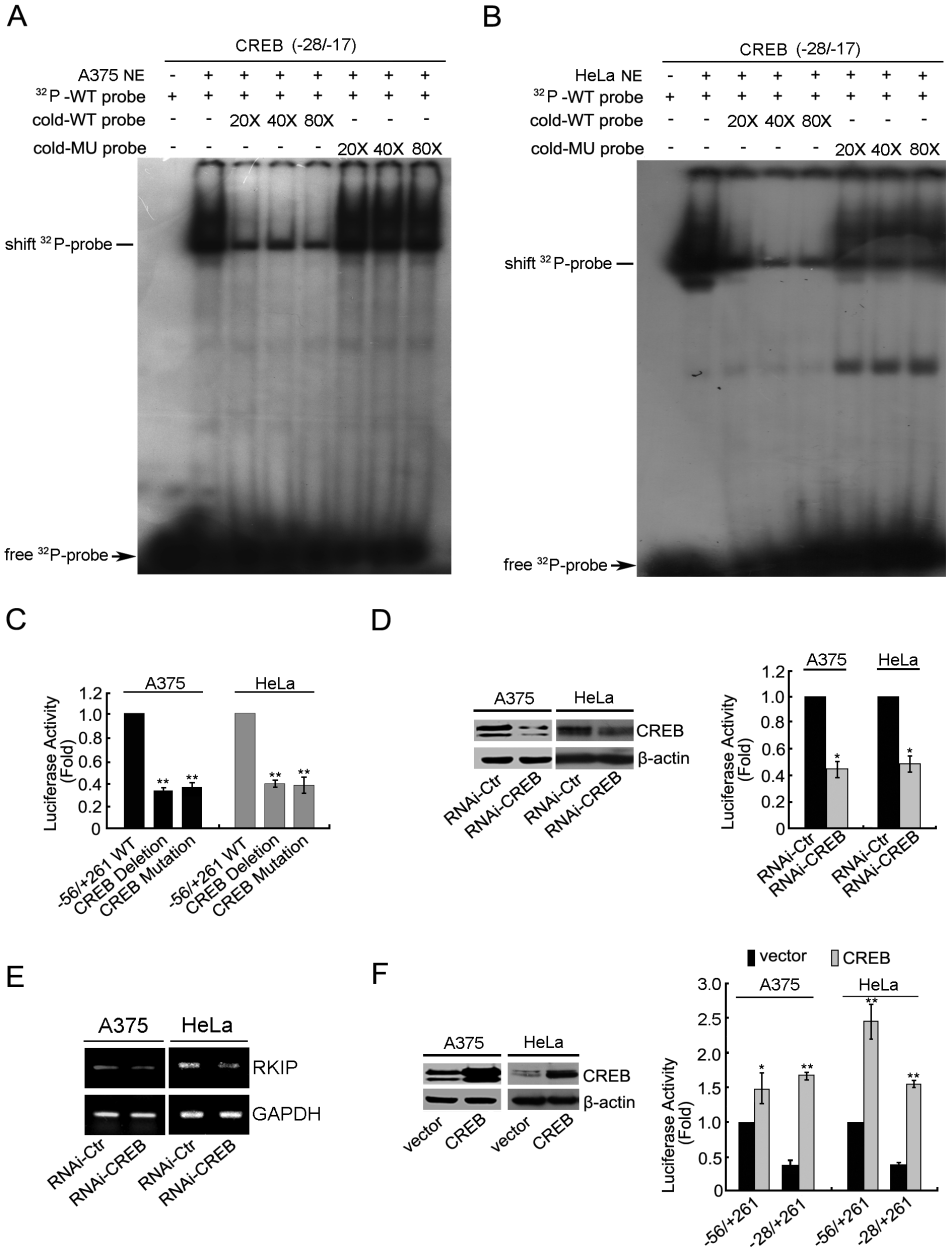
Role of CREB binding in regulating RKIP promoter activity in A375 and HeLa cells. In A375 (A) and HeLa (B) nuclear extracts, EMSA of the ^32^P-labeled oligonucleotide derived from the −28/−17 putative CREB binding site in the presence or absence of cold WT or mutation oligonucleotide. NE, nuclear extract. Shifted ^32^P-probe bands sensitive to competition by the WT cold probe are indicated. (C) Relative luciferase activity of cells transfected with each of the indicated reporter constructs. (D) Cells were co-transfected with a negative control or a CREB-specific shRNA and the reporter construct pGL3-Basic RKIP (−56/+261), then cultured for an additional 24 hr. Left panel: immunoblots of cell lysates. Right panel: relative luciferase activity. (E) Cells were transfected with the indicated siRNAs and then the mRNA level of RKIP was examined by RT-PCR. (F) Cells were co-transfected with the vector (pcDNA 3.1) or the CREB expression plasmid and the reporter construct, then cultured for an additional 24 hr. Left panel: immunoblots of cell lysates. Right panel: relative luciferase activity. *P<0.05, **P<0.01.

Binding of CREB to a nearby region of the transcription start site often enhances the assembly of a functional transcription complex through direct interaction with both TFIIB and TFIID [20]. To define the role of the putative CREB binding site in regulating RKIP promoter activity, we synthesized a pair of oligonucleotides according to the putative CREB bindingsite sequence and determined its interaction with factors in nuclear extracts from A375 or HeLa cells by EMSA. For both cell lines, incubation of the ^32^P-labeled oligonucleotide with nuclear extracts resulted in one obvious gel mobility shift band. The shift band was greatly reduced by adding a large excess (20- to 80-fold) of the cold WT oligonucleotide but not by adding the same concentration of the mutant oligonucleotide (Fig. 4A–B). We then mutated or deleted the putative CREB binding site in the reporter construct containing the region −56 to +261 and determined the effect on promoter activity by the luciferase assay. In both A375 and HeLa cells, both the mutated and the deleted CREB binding sites resulted in an ∼60% reduction in luciferase activity (Fig. 4C). These results demonstrate that the putative CREB binding site plays a positive role in RKIP promoter activity.

To determine whether the CREB binding site contributes to RKIP promoter activity by recruiting CREB, we transfected A375 and HeLa cells with CREB-specific shRNA and determined the effect of CREB knockdown on both the promoter activity of the reporter construct and the level of the endogenous RKIP transcript. In both cell lines, transfection of CREB-specific shRNA reduced the expression of CREB to ∼40% of its original level (Fig. 4D, left panel), which resulted in an ∼50% reduction in promoter activity (Fig. 4D, right panel) and an ∼40% reduction in the level of RKIP transcript (Fig. 4E). We also examined the effects of overexpression of CREB on the RKIP promoter activity of two different reporter constructs (region −56 to +261 and region −28 to +261). In contrast to the effect of CREB knockdown, CREB overexpression increased the luciferase activity of both constructs (Fig. 4F). For both cell lines, the effect appeared to be greater on the −28/+261 construct than on the −56 to +261 construct. These results support the notion that, through its interaction with the CREB binding site, CREB is a major transcription factor that promotes RKIP transcription.

### Roles of p300 Binding in Regulating RKIP Promoter Activity

**Figure 5:**
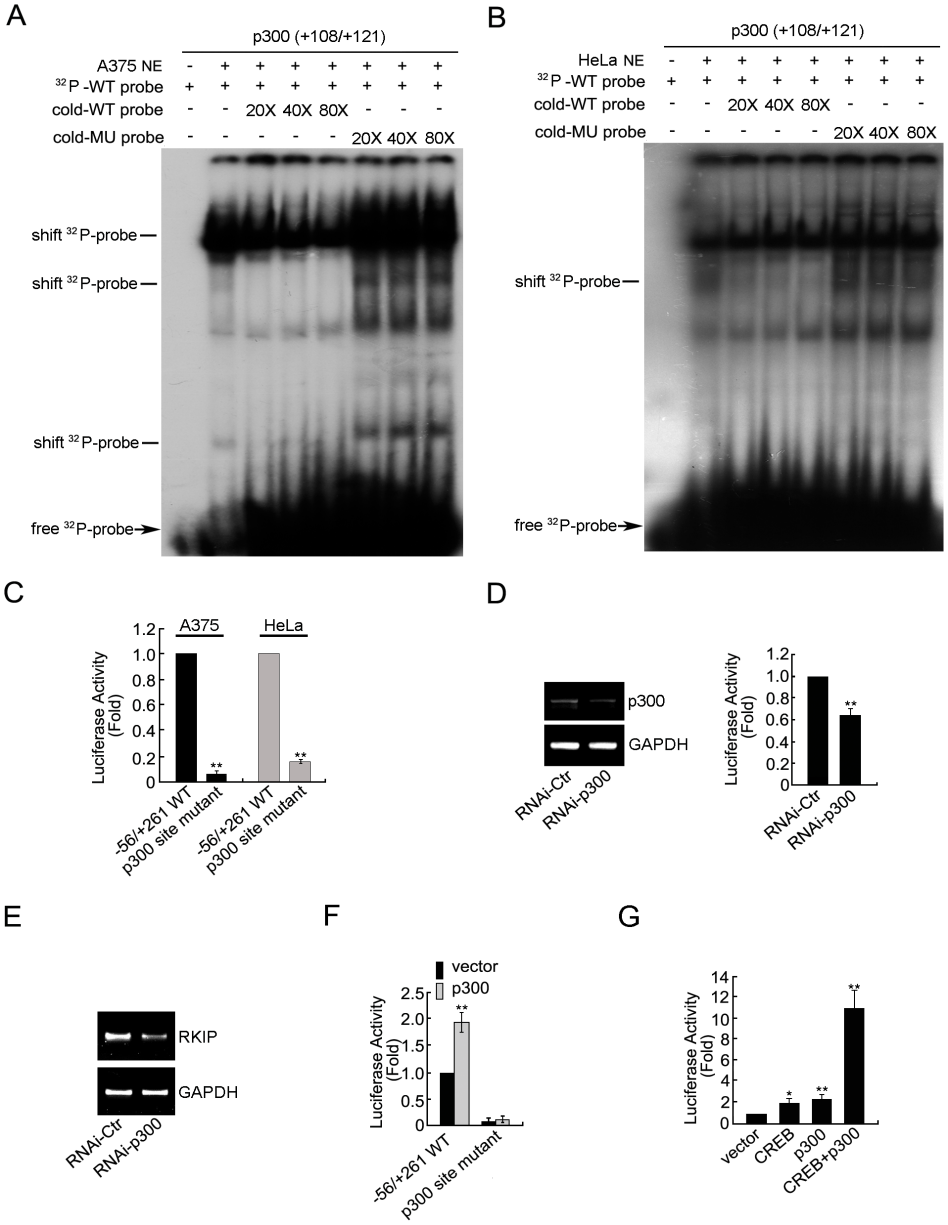
Roles of p300 binding in regulating RKIP promoter activityin A375 and HeLa cells. In A375 (A) and HeLa (B) nuclear extracts, EMSA of the ^32^P-labeled oligonucleotide derived from the +108/+121 putative p300 binding site in the presence or absence of cold WT or mutation oligonucleotide. NE, nuclear extract. Shifted ^32^P-probe bands sensitive to competition by the WT cold probe are indicated. (C) Relative luciferase activity of A375 and HeLa cells transfected with the indicated reporter constructs. (D) A375 cells were co-transfected with negative control or p300-specific siRNA and the reporter construct pGL3-Basic RKIP (−56/+261), then cultured for an additional 48 hr. Left panel: RT-PCR. Right panel: relative luciferase activity. (E) A375 cells were transfected with the indicated siRNAs and the mRNA level of RKIP was examined by RT-PCR. (F) Relative luciferase activity of A375 cells co-transfected as indicated and cultured for an additional 48 hr. (G) A375 cells were co-transfected with the reporter construct pGL3-Basic RKIP (−56/+261) and the indicated plasmids and then cultured for an additional 48 hr. Relative luciferase activity was assayed. *P<0.05, **P<0.01.

The well-characterized acetyltransferase p300 increases the rate of transcription by loosening nucleosomal structures [21]. To determine the role of the putative p300 binding site (+108 to +121) in RKIP promoter activity, we first synthesized a pair of oligonucleotides according to this putative site sequence and determined its interaction with nuclear factors from A375 or HeLa cells by EMSA. For both cell lines, incubation of the ^32^P-labeled oligonucleotide with nuclear extracts generated three large band shifts, and the second band was almost eliminated by a 20-fold increase of the WT cold oligonucleotide but was not notably affected by the mutant oligonucleotide (Fig. 5A–B). However, the first band behaved differently in the two cell lines: in A375 cells this band was specifically and moderately reduced with cold WT oligonucleotide in a dose-dependent manner, whereas in HeLa cells the band was not sensitive to competition by the cold WT oligonucleotide. Regardless of the difference observed in the two cell lines, these results indicate that the p300 binding site interacts with nuclear factors.

Because p300 promotes transcription through changing chromatin structures, transfected plasmid DNA acquires regular nucleoprotein structures in the nucleus, and the transcription of plasmid DNA is carried out by the same cellular mechanisms that carry out the transcription of endogenous genes [22], plasmid DNA transfection and luciferase assay are suitable for determining the functional impact of the interaction between the p300 binding site and nuclear factors. Therefore, we mutated the p300 binding site in the reporter construct and determined the effect on promoter activity in A375 and HeLa cells by luciferase assay. In both cell lines, mutation of the p300 binding site resulted in a >80% reduction in luciferase activity (Fig. 5C), demonstrating that the p300 binding site plays a positive role in promoter activity in both cell lines.

To determine the involvement of p300 in RKIP transcription, we examined the effect of p300 knockdown or overexpression on RKIP promoter activity in A375 cells. Transfection of A375 cells with p300-specific siRNA caused an ∼50% reduction in the mRNA level of p300 (Fig. 5D, left panel), which resulted in an ∼40% reduction in the promoter activity of the reporter construct (Fig. 5D, right panel) and an ∼50% reduction in the RKIP transcript (Fig. 5E). In contrast, transfection of A375 cells with a p300 expression plasmid caused a 2-fold increase in promoter activity, but the stimulatory effect was eliminated by mutation of the p300 binding site in the reporter construct (Fig. 5F). Together, these results demonstrate that through its interaction with the p300 binding site, p300 is one of the major transcription factors that promote RKIP transcription.

### Synergistic Effects of CREB and p300 on RKIP Promoter Activity in A375 Cells

Previous studies demonstrated that p300 interacts and synergizes with CREB to promote transcription [23], [24]. To determine whether this is the case in RKIP promoter activity regulation by p300 and CREB, we ectopically expressed p300 and CREB alone or together in A375 cells and determined the effect on RKIP promoter activity. Expression of CREB increased RKIP promoter activity by 1.9-fold and expression of p300 increased it by 2.2-fold; simultaneous expression of both proteins increased it by >10-fold (Fig. 5G). These results indicate that p300 synergize with CREB to enhance RKIP promoter activity in A375 cells.

## Discussion

In this study, we demonstrated that the region −56 to +261 in the RKIP gene exhibits full promoter activity. Within this region there are two Sp1 binding sites (region −17 to −6 and region −5 to +5), one functional CREB binding site upstream of the Sp1 binding sites (region −28 to −17), and one p300 binding site downstream of the Sp1 binding sites (region +108 to +121). All three kinds of *cis*-acting elements and the corresponding transcription factors were found to play positive roles in RKIP promoter activity. These results expand upon the previous finding by Okita et al that the region −97 to +1 in the RKIP gene is important for RKIP transcription and provide a solid basis for further investigation of the regulation of RKIP expression in physiological and pathological processes [14]. In addition to these defined positive regulators, we found that the region −813 to −193 in the RKIP gene negatively regulated RKIP promoter activity, indicating that this region contains *cis*-acting elements that interact with transcription suppressors. Sequence analysis of this region by bioinformatics revealed the presence of putative binding sites for the transcription factors AML-1a, Thing1/E47, CdxA, GATA1/GATA2, ELK-1, IK-2, and Lyf-1. Whether any of these sites is involved in recruiting negative regulators of RKIP transcription is a subject for additional studies.

A minimal promoter activity requires the assembly of general transcription machinery by binding of an initiating protein near the transcription initiation site [25]. Assembly can be achieved through TATA box-mediated and non-TATA box-mediated mechanisms [26]. Binding of the TATA box binding protein to the TATA box and of co-factors such as TFIIA and TFIIB to nearby sites leads to sequential assembly of the general transcription machinery (e.g., TFIIE, TFIID, TFIIF, TFIIH, Pol II) and, as a result, transcription begins [27], [28]. In promoters lacking a classical TATA box or an analogous site (e.g., initiator), binding of Sp1 to sites with high G/C content directs the formation of the transcription initiation complex through slightly modified mechanisms [29]. We found that although the minimal promoter region of RKIP does not contain a TATA box or an analogous site, it contains two adjacent functional Sp1 sites which both have functions. Thus, it seems that the assembly of the general transcription machinery for RKIP transcription is achieved through a Sp1-dependent mechanism. A compensatory effect of Sp1 may take place within the two Sp1 sites, which could account for our observation that a single site mutation caused only a moderate decrease in RKIP transcription.

The efficiency of the assembly of the general transcription machinery is greatly enhanced by recruitment of co-activators that both bind to regions close to the minimal promoter region and interact with components in the general transcription machinery [25]. A previous study demonstrated that one such co-activator, CREB, functions through directly interacting with both TFIIB and TFIID [20]. Our results showed that the region –28 to –17 is a functional CREB binding site. Elimination or mutation of this site or knockdown of CREB expression greatly reduced but did not eliminate RKIP promoter activity. We speculate that interaction of CREB with the RKIP promoter enhances the Sp1-dependent assembly of the general transcription machinery that conducts RKIP transcription.

The rate of the transcription conducted by the assembled transcription machinery is known to be greatly affected by chromatin structures [30]. Highly packed chromatin structures are not conducive to the passage of the general transcription machinery, so factors that loosen nucleosomal structures enhance the rate of transcription [31]. Because histone acetylation de-condenses nucleosomal structures, recruitment of an acetyltransferase to a nearby promoter region often enhances the rate of transcription. Previous studies have demonstrated that p300 is one of the well-characterized acetyltransferases that promote transcription through acetylating histone tails [21], [32]. In this study, we present evidence that both the p300 binding site and p300 expression play a positive role in RKIP promoter activity and that p300 overexpression synergizes with CREB overexpression to enhance RKIP promoter activity in A375 cells. These findings led us to hypothesize that p300-mediated histone acetylation promotes RKIP transcription through de-condensing the chromatin structure of the RKIP gene.

Identification of these three kinds of transcription factors that positively regulate the RKIP promoter activity suggests that the expression or function of these transcription factors regulates RKIP expression. Thus far, the transcriptional activity and stability of Sp1 can be regulated by posttranslational modifications, including phosphorylation, acetylation, sumoylation, ubiquitylation and glycosylation [33]. Phospho-regulation of Sp1 involves multiple protein kinases, including CDK, PKC-ζ, ERK, casein kinase II, and DNA-dependent protein kinase [33]. CREB can be activated through phosphorylation by a number of kinases, including Akt, p90Rsk, protein kinase A, and calcium- or calmodulin-dependent kinases [34], and p300 is regulated by PKC, AMPK, and Akt [35]–[37]. It is conceivable that the dysregulation of some of these signaling pathways is responsible for the downregulation of RKIP in pathological processes such as cancer and Alzheimer’s disease.

## Materials and Methods

### Cell Culture

The A375 (CRL-1619) and HeLa (CCL-2) cell lines were purchased from American Type Culture Collection and cultured at 37°C in 5% CO_2_ in Dulbecco’s modified Eagle’s medium (GIBCO) supplemented with 10% fetal bovine serum (HyClone).

### Genomic PCR and Gene Manipulation

Genomic PCR was performed by using Phusion high-fidelity DNA polymerase (New England Biolabs) and human genomic DNA (Promega) as the template. The RKIP gene transcription regulatory region was cloned into the pGL3 Basic vector (Promega), and various deletion constructs were prepared by PCR with added *Kpn* I and *Bgl* II sites for directed cloning. Site-directed mutations were introduced using mutant primers (Table S1). All PCR-amplified fragments and mutation constructs were verified by DNA sequencing. All restriction enzymes were purchased from Takara.

siRNAs used for silencing endogenous expression of Sp1 and p300 were synthesized by GenePharma according to the sequence information previously reported [38], [39]. The CREB-specific shRNA plasmid and a negative control plasmid were purchased from Upstate.

Generation of CREB plasmid was engineered using a PCR technique. We amplified a 1059-bp fragment of the human genomic cDNA and reverse-transcripted it to mRNA by using M-MLV reverse transcriptase (Promega) according to the manufacturer’s protocol. The following primers were used for amplification: 5′-CGGAATTCGGTAACTAAATGACCA-3′ (sense, containing the *Eco*R I restriction site) and 5′-CGGGATCCATCCCAAATTAATCT G-3′ (anti-sense, containing the *Bam*H I restriction site). The PCR products were inserted into the pcDNA3.1 (-) vector (Invitrogen) and the construct was confirmed by DNA sequencing.

### Transient Transfection and Luciferase Assay

Cells were plated at a density of 1.6×10^5^ cells/well in 24-well plates. On the following day, transfection was performed with 0.6 µg of the indicated promoter-luciferase constructs or the empty vector pGL3-Basic together with 0.2 µg of pCMV-β-galactosidase plasmid. Transfection was performed using Lipofectamine2000 transfection reagent (Invitrogen) according to the manufacturer’s instructions. For RNAi experiments, specific siRNA or control RNA was co-transfected into A375 or HeLa cells with the reporter plasmids respectively, and the cells were further cultured for 48 hr. Cells were then lysed with Promega’s cell culture lysis reagent (25 mM Tris-phosphate, 2 mM DTT, 2 mM 1,2-diaminocyclohexane-*N,N,N′,N′*-tetraacetic acid, 10% glycerol, 1% Triton X-100, 1.25 mg/ml lysozyme, 2.5 mg/ml BSA), and luciferase activity level was measured using Promega’s luciferase assay system (Cat.#E1500) and normalized to β-galactosidase activity [40].

### Immunoblotting

Protein extraction from A375 or HeLa cells, SDS-PAGE, and immunoblotting were performed as previously described [41]. The primary antibodies against Sp1 and CREB were obtained from ABGENT (AP11451b and AP11707c), the primary antibody against p300 was purchased from Assay Biotech (C0289), and the primary antibody against actin and the horseradish peroxidase-conjugated goat anti-rabbit or goat anti-mouse antibodies were obtained from Santa Cruz Biotechnology (sc-69879, sc-2004, and sc-2005). Immunoblots were developed with enhanced chemiluminescent reagents from Amersham.

### EMSA

Nuclear extracts were prepared by using a Fermentas cytoplasmic and nuclear protein extraction kit. Complementary strands of oligonucleotides were synthesized by Invitrogen (Table S2). EMSA was carried out using the Promega gel shift assay system. Briefly, the binding probes and their complementary pairs were incubated at 70°C for 10 min in annealing buffer (100 mM NaCl, 1 mM EDTA, and 10 mM Tris·HCl pH 7.5) and allowed to cool down slowly to room temperature. Double-stranded oligonucleotides were then end-labeled with [γ-^32^P] ATP. Nuclear extracts were incubated with ^32^P-labeled oligonucleotide probes with or without unlabeled oligonucleotide competitors (20- to 80-fold molar excess of labeled probes). The samples were then separated with 4% non-denaturing polyacrylamide gel electrophoresis at 300 V in 0.5×TBE buffer for 2 hr. The gel was dried and exposed to X-ray film (Kodak) overnight at −80°C.

### RT-PCR

Total RNA was extracted from A375 or HeLa cells using TRIZOL reagent (Invitrogen) according to the manufacturer’s protocols. For reverse transcription (RT), 2 µg of RNA was annealed to oligo (dT) at 65°C for 5 min and cooled to room temperature. The RNA-oligo (dT) mixture was then incubated with M-MLV reverse transcriptase (Promega) and dNTPs at 42°C for 1 hr. For the PCR step, RT products were amplified by PCR under log phase conditions. The sequences of the primers for RT-PCR were as follows: RKIP, 5′-AGACCCACCAGCATTTCGTG-3′ and 5′-GCTGATGTCATTGCCCTTCA-3′; p300, 5′-AGGTCTTCTTTGTGATCCG-3′ and 5′-CCAACCACACCAGTCCG-3′; GAPDH, 5′-GCACCGTCAAGGCTGAGAAC-3′ and 5′-TGGTGAAGACGCCAGTGGA-3′.

### Bioinformatics

Analysis of the transcription factor binding sites was done by TFSEARCH online (www.cbrc.jp/research/db/TFSEARCH.html) using the default parameters.

### Statistical Analysis

Data are expressed as mean ± SEM. Differences between experimental groups were assessed using the Student t-test. A P value <0.05 was considered statistically significant.

## Supporting Information

Table S1 PCR primers for mutation.
(DOC)

Table S2 Oligonucleotides for EMSA.
(DOC)
