# Supplementary material for: cis-Acting Elements and trans-Acting Factors in the Transcriptional Regulation of Raf Kinase Inhibitory Protein Expression
Source: PLoS One. 2013 Dec 26;8(12):e83097. doi: 10.1371/journal.pone.0083097 (PMC3873293; doi:10.1371/journal.pone.0083097)
Supplement: Table S1 — PCR primers for mutation. (DOC) [file pone.0083097.s001.doc]

**Table S1. PCR primers for mutation**

| **Primer name** | **Forward primer** | **Reverse primer** | **Mutation sites** |
| --- | --- | --- | --- |
| p300M | 5’-CTACCGCGGCAGAGTCGGCT  GCAC-3’ | 5’-GTGCAGCCGACTCTGCCGCG  GTAG-3’ | WT: 5’ -GCGGCACTCCCGGC-3’  MT: 5’ -GCGGCAGAGTCGGC-3’ |
| Sp1 (-17/-6) M | 5’-GGGAATTCCGCGGGGC-3’ | 5’-CGGAATTCCCACGTCACGC-3’ | WT: 5’ -GGGCGGTGCGC-3’  MT: 5’ -GGGAATTCCGC- 3’ |
| Sp1 (-5/+5) M | 5’-GGGAATTCGCGGCGGCTGA-3’ | 5’-GCGAATTCCCGCGCACCGC-3’ | WT: 5’ -GGGGCTGGGCGGCGG-3’  MT: 5’ -GGGAATTCGCGGCGG-3’ |
| CREBM | 5’-GTGAATTCGGGCGGTGCGCG-3’ | 5’-CCGAATTCACGCCCACCGCT  C-3’ | WT: 5’ -CGTGACGTGGGGCGG-3’  MT: 5’ -CGTGAATTCGGGCGG-3’ |

WT, wild type; MT, mutation.
