# Supplementary material for: cis-Acting Elements and trans-Acting Factors in the Transcriptional Regulation of Raf Kinase Inhibitory Protein Expression
Source: PLoS One. 2013 Dec 26;8(12):e83097. doi: 10.1371/journal.pone.0083097 (PMC3873293; doi:10.1371/journal.pone.0083097)
Supplement: Table S2 — Oligonucleotides for EMSA. (DOC) [file pone.0083097.s002.doc]

**Table S2. Oligonucleotides for EMSA**

| **Transcription factor** | **Forward primer** | **Reverse primer** |
| --- | --- | --- |
| CREB | 5’-TGGGCGTGACGTGGGGCGGT-3’ | 5’-ACCGCCCCACGTCACGCCCA-3’ |
| CREB mutation | 5’-TGGGCGAGAATTCGGGCGGT-3’ | 5’-ACCGCCCGAATTCTCGCCCA-3’ |
| Sp1 I | 5’-GACGTGGGGCGGTGCGCGGGG-3’ | 5’-CCCCGCGCACCGCCCCACGTC-3’ |
| Sp1 I mutation | 5’-GACGTGGAGGAATTCGCGGGG-3’ | 5’-CCCCGCGAATTCCTCCACGTC-3’ |
| Sp1 II | 5’-GTGCGCGGGGCTGGGCGGCGGC-3’ | 5’-GCCGCCGCCCAGCCCCGCGCAC-3’ |
| Sp1 II mutation | 5’-GTGCGAGAGAATTCGCGGCGGC-3’ | 5’-GCCGCCGCGAATTCTCTCGCAC-3’ |
| p300 | 5’-CCGCGGCACTCCCGGCTGC-3’ | 5’-GCAGCCGGGAGTGCCGCGG-3’ |
| p300 mutation | 5’-CCGCGGAATTCACGGCTGC-3’ | 5’-GCAGCCGTGAATTCCGCGG-3’ |
